# Iron Oxide Nanoparticles for Biomedical Applications: A Perspective on Synthesis, Drugs, Antimicrobial Activity, and Toxicity

**DOI:** 10.3390/antibiotics7020046

**Published:** 2018-06-09

**Authors:** Laís Salomão Arias, Juliano Pelim Pessan, Ana Paula Miranda Vieira, Taynara Maria Toito de Lima, Alberto Carlos Botazzo Delbem, Douglas Roberto Monteiro

**Affiliations:** 1 Department of Pediatric Dentistry and Public Health, School of Dentistry, Araçatuba, São Paulo State University (Unesp), 16015-050 Araçatuba/São Paulo, Brazil; laisarias@hotmail.com (L.S.A.); jpessan@foa.unesp.br (J.P.P.); anapaula.mvieira@hotmail.com (A.P.M.V.); adelbem@foa.unesp.br (A.C.B.D.); 2 Graduate Program in Dentistry (GPD-Master’s Degree), University of Western São Paulo (UNOESTE), 19050-920 Presidente Prudente/São Paulo, Brazil; taynaramaria@hotmail.com

**Keywords:** biotechnology, drug delivery, iron oxide nanoparticles, magnetic nanoparticles

## Abstract

Medical applications and biotechnological advances, including magnetic resonance imaging, cell separation and detection, tissue repair, magnetic hyperthermia and drug delivery, have strongly benefited from employing iron oxide nanoparticles (IONPs) due to their remarkable properties, such as superparamagnetism, size and possibility of receiving a biocompatible coating. Ongoing research efforts focus on reducing drug concentration, toxicity, and other side effects, while increasing efficacy of IONPs-based treatments. This review highlights the methods of synthesis and presents the most recent reports in the literature regarding advances in drug delivery using IONPs-based systems, as well as their antimicrobial activity against different microorganisms. Furthermore, the toxicity of IONPs alone and constituting nanosystems is also addressed.

## 1. Introduction

The development of nanotechnology has provided resources to various applications in the medical field, leading to significant advances in terms of diagnosis, biological detection, therapy and drug delivery [1,2,3,4,5]. In this context, magnetic nanoparticles comprise important characteristics that make them attractive for a variety of biomedical applications, including contrast agents in magnetic resonance imagining (MRI) [6], cell separation and detection [7,8], treatment for hyperthermia [9] and drug delivery [10]. Specifically, iron oxide magnetic nanoparticles (IONPs) are physically and chemically stable, biocompatible and environmentally safe [11], thus presenting unique characteristics for clinical applications. However, when IONPs (Fe_3_O_4_ (magnetite) or γ-Fe_2_O_3_ (maghemite)) reach smaller sizes (about 10–20 nm for iron oxide), superparamagnetic properties become evident, so that the particles reach a better performance for most of the aforementioned applications [11,12].

Despite the growing body of evidence attesting their biomedical usefulness, superparamagnetic IONPs are still in early stage of clinical investigation, with studies pointing out to the need for their improvement prior to their commercialization. Most of clinical trials with IONPs have been developed within the last decade, being MRI imaging the main application assessed [13,14]. The number of clinical trials indexed on *clinicaltrials.gov* [14] under the term ‘iron oxide nanoparticles’ comprises fourteen protocols. Of these, four are completed, four are still active, one was withdrawn, four were suspended or terminated, and one has an unknown status. Published data from one of those clinical trials showed that IONPs succeeded to act as contrast agent for MRI for the assessment of cellular myocardial inflammation following acute myocardial infarction, with no described adverse effects to the patients [15].

Issues related to biocompatibility, toxicological and immunological parameters are other challenges that need to be addressed. Data on methods of synthesis show that IONPs functions are directly related to size, shape, coating and stability of these nanoparticles [16]. As an example, large nanoparticles (>200 nm) are easily cleared by the reticuloendothelial system [17,18], while particles smaller than 10 nm are easily excreted from the body through existent pores of the kidney’s basal lamina [19], what reduces their blood-circulating time. Further, hydrophobic and negatively charged nanoparticles tend to suffer proteic opsonization and are quickly recognized by phagocytic cells [20], also resulting in faster clearance. These and other IONPs limitations, such as oxidation and cell toxicity, can be overcome by an adequate surface-coating, implying that the success of a IONPs-based nanosystem is also directly related to the properties of the coating material. Different organic and inorganic coatings, including natural and synthetic polymers [21,22,23], surfactants [24], gold [25], silica [26] and peptides [27] have been investigated in studies showing that shape, spatial configuration and nature of the coating play an important role on the nanosystem’s performance.

This review provides conceptual information on methods of IONPs synthesis, addressing the main advantages and disadvantages, and drugs bound to IONPs in the production of drug-delivery nanosystems. The latest updates on bioapplications, translational advances, and the employment of IONPs on antimicrobial therapeutic alternatives are also covered, bringing new perspectives on IONPs investigations. Finally, a set of considerations is made on IONPs toxicological aspects, as well as advances on coating strategies to elaborate more biocompatible nanosystems.

## 2. Synthesis of IONPs

There are three main routes for the synthesis of IONPs: chemical, physical and biological. These have been investigated in order to produce more stable, soluble, biocompatible, and shape and size-controlled nanoparticles [28]. This review presents an overview on the most common methods of synthesis, highlighting advantages and disadvantages of each method.

### 2.1. Chemical Routes

#### 2.1.1. Co-Precipitation

Among the chemical methods of synthesis of IONPs, the aqueous co-precipitation is the most commonly used [18,29]. Shortly, salts of Fe^2+^ and Fe^3+^ ions suffer co-precipitation in a fairly basic solution (molar ratio 1:2) at room temperature or under heat [29,30,31,32]. In general, this is a convenient and low cost method that enables rapid large-scale production. However, the resulting nanoparticles present problems of aggregation and large size distribution, which is common in aqueous routes [33], in addition to poor crystallinity and tendency to oxidize, thus compromising their magnetic properties [18,30].

Given that base concentration, temperature, Fe^2+^/Fe^3+^ proportion, value and ionic strength of the media, order of the reactants and the use of surfactants are factors that may interfere with the control of particle size, shape, composition and magnetic properties [34,35], recent studies have adapted the co-precipitation method in order to improve the properties of the nanoparticles [36,37,38,39]. For instance, through variations in the pH of the precipitates and in the amount of sodium hydroxide, spherical IONPs of different sizes can be obtained [40], taking advantage of the linear relation between IONPs diameter and pH, probably due to nanoparticle aggregation.

#### 2.1.2. Microemulsion

The microemulsion method acts confining the production of nanoparticles inside a nanosystem that combines a stable isotropic mixture of oil and water, whose interface is stabilized by a monolayer of surfactant, sometimes combined with a co-surfactant [31,41,42]. The size of the microemulsion, whether direct (oil dispersed in water) or indirect (water dispersed in oil), may be controlled by adjusting the ratio of water, oil and surfactant, which consequently leads to the IONPs size control [29,43,44]. Microemulsion experiments demonstrated that the nature of the surfactant, concentration of Fe^2+^/Fe^3+^ ions, as well as the temperature and the pH value strongly influence the nanoparticles size distribution and, consequently, their magnetization [33]. Despite the narrow size distribution that this method provides, it presents certain limitations for biomedical purposes, including requirements of low temperatures and large quantity of oil, which limits large scale production. Moreover, surfactants adhered to IONPs are difficult to remove [17,31,42].

#### 2.1.3. Hydrothermal and Solvothermal Syntheses

Through the hydrothermal method, iron precursors are exposed to vapor in a sealed container, under high pressure and temperature conditions in an aqueous medium, creating uniform IONPs [17]. Under these conditions, the controlled oxidation of Fe_3_O_4_ and the mineralization of Fe^3+^ ions occur. When the aqueous synthesis methods generate particles with low crystallization, the solvothermal method can be used, which consists in replacing water by other organic solvents, allowing the formation of monodisperse IONPs with high crystallinity and controlled shape [28,35,45,46]. However, such methods last much longer (hours to days) in comparison to the microemulsion method [11,29].

#### 2.1.4. Thermal Decomposition

The thermal decomposition is a non-aqueous synthesis in which organometallic compounds such as Fe(Acac)_3_, Fe(C_2_O_4_) × 2H_2_O, Fe(CH_3_COO)_2_ or ferrocene suffer decomposition in a high-boiling organic solvent (or via solvent free) in the presence of stabilizing surfactants like aliphatic amine and fatty acids [30,47]. This method circumvents the limitations of co-precipitation by generating high quality IONPs with close distribution and particle size control, as well as improving their magnetism and degree of crystallinity [30,47,48]. This occurs due to the possibility of separating the nucleation from the growth, avoiding complex reactions of hydrolysis [29,49]. Iron oxide nanocrystals with narrow size distributions, high yield and without aggregation can be obtained by this method [50]. However, as the resulting IONPs are insoluble in water, further steps are required to make their surfaces hydrophilic and, consequently, compatible with biological solutions [18,51]. Also, a higher relaxivity and saturation magnetization can be achieved by higher temperatures and shorter synthesis reactions [52].

#### 2.1.5. Sol-Gel Reaction and Polyol

The sol-gel reaction is a wet-chemical method in which iron alkoxides and salts (e.g., chlorides, nitrates and acetates) undergo reactions of condensation and hydrolysis [29,53]. Factors implicated in the process such as pH, temperature and concentration of the reagents can interfere with the final crystallinity of the product. The main advantage of this method is the production of structures with good homogeneity and size, and high purity and quantity. However, precursors used in the reaction are costly, and the resulting nanoparticles may display high permeability and low wear resistance [54,55]. On the other hand, the polyol method uses a reduction reaction in which the polyol is heated to its boiling point, and acts as a solvent and reducing agent in a medium with iron precursors, controlling the growth of the particles [56]. Noteworthy, the size of the nanoparticles varies according with the polyol used, reaction time and concentration of iron precursor. In this sense, the size of the nanoparticles was shown to increase proportionally to the length of the glycol [57].

#### 2.1.6. Sonochemical

In sonochemical synthesis, a ferrous salt solution is subjected to a high-intensity ultrasonication at room temperature, ensuring that the physical effects generated from the creation of blistering in solution generates the energy required for the reaction [16,28]. This method can be an unusual and versatile alternative for materials synthesis owing to short reaction times, and lack of high process temperature and pressure. As an example, the highest percentage of atomic concentration when functionalizing 3-amino propyl triethoxyl silane with IONPs was achieved after one minute of sonication [58]. In addition, the ultrasonic frequency may play a role in the products generated by this method, considering that the amount of the drug loaded proportionally increases with ultrasound frequency [59].

#### 2.1.7. Microwave-Assisted Synthesis

Microwave-assisted synthesis is a relatively simple and recent method, in which a mixture containing the iron precursors is exposed to microwave electromagnetic radiation, causing molecule reorientation, and strong and homogeneous internal heating [16,29]. This method has several advantages such as low-cost, reduced time of reaction and narrow size- and shape-control of the IONPs [29,35], which make it attractive for cost-efficient commercial production of IONPs with appropriate solubility and biocompatibility for clinical trials [60].

### 2.2. Physical Routes

#### 2.2.1. Pyrolysis Method

In pyrolysis method, gaseous (aerosol) organometallic precursors are exposed to laser radiation that transmits energy in a selective way, according to the chemicals wavelength, thus generating IONPs [61]. This route produces pure and homogeneous samples with good size control and shape distribution. It is less expensive than other methods, since it operates at atmospheric pressure [35,62]. Factors such as precursors concentration, working pressure and laser intensity directly interfere with size and magnetization of the resulting nanoparticles [63].

#### 2.2.2. Laser Ablation Synthesis in Solution (LASiS)

This synthesis is triggered when a pulsed laser beam reaches the target material immersed in liquid solution, causing changes in the composition of the ablation target and in the liquid solution [64]. Although LASiS is an interesting technique for materials of different structures and compositions, this method carries problems depending on the solvent used, such as the difficulty of controlling particle size and their clustering [64]. Recent experiments, however, revealed that the use of laser ablation on phosphonates aqueous solution and bulk iron was successful in decreasing the size of FeO_x_ crystal to few atom clusters [65].

### 2.3. Biological Route

#### Biosynthesis

Biosynthesis stands out as a simple, low-cost and eco-friendly route of IONPs production [66], in which plant extracts or microbial-derived products with reducing potential interact with iron precursors under stirring. The main reaction involved in this process is reduction/oxidation, and the resulting nanoparticles usually display good biocompatibility [16,29,66]. The IONPs biosynthesis can use iron-reducing bacteria such as *Geobacter metallireducens* [67] but may include many other possibilities. One example is the synthesis by the enzyme lumazine synthase (produced both by fungi and bacteria), which serves as a biological nanoreactor, synthesizing IONPs inside narrow-diameter capsid templates [68].

## 3. IONPs Coating and Functionalization

**Figure 1:**
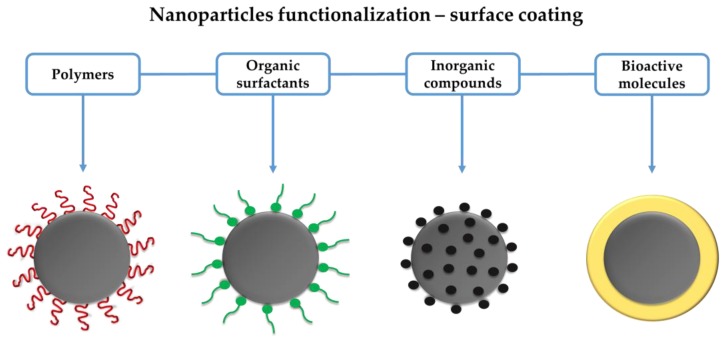
Schematic illustration of the main shells for functionalization of iron oxide nanoparticles (IONPs). Grey circles represent the core of IONPs.

Core-shell nanosystems are often employed to attach different drugs to IONPs. The nanoparticle corresponds to the core, while shell represents the surface coating for nanoparticle functionalization, improving its stability, pharmacokinetics, biodistribution and biocompatibility [69]. Synthetic and natural polymers, organic surfactants, inorganic compounds and bioactive molecules can function as shell of IONPs, as summarized in Figure 1.

### 3.1. Synthetic and Natural Polymers

Polymers are the most common surface coating used in IONPs, since they can prevent oxidation and confer stability to the nanoparticles [70]. The polymer nature can be synthetic, encompassing polyethylene glycol, poly(vinylpyrrolidone), polyvinyl alcohol and poly(lactic-co-glycolic acid) [28,29,35], or natural, as in the case of chitosan.

#### 3.1.1. Polyethylene Glycol (PEG)

PEG is a hydrophilic, uncharged polyether polymer well known for its biocompatibility [71]. It has been commonly used as IONPs-coating due to non-fouling properties, reduced blood proteins opsonization and, as a result, escapes recognition by the immune system. Such properties increase its time in blood circulation and the accumulation in the target cells/organ [28,70,71].

#### 3.1.2. Poly(vinylpyrrolidone) (PVP) e Polyvinyl Alcohool (PVA)

PVP e PVA are water-soluble synthetic polymers. Specifically, PVP is derived from the monomer N-vinyl pyrrolidone [72]. Given its biocompatible, stable and safe properties, PVP is widely used in biomedical and pharmaceutical applications [72,73]. In turn, PVA exhibits emulsifying and adhesive properties, forming a hydrogel structure that involves the IONPs by means of hydrogen bonds between the polymer chains. This leads to increases in polymer-surface interactions, preventing the agglomeration of the particles [70,73].

#### 3.1.3. Poly(lactic-*co*-glycolic acid) (PLGA)

PLGA is a copolymer of poly lactic acid and poly glycolic acid (PGA) [74] with great potential for use in drug delivery and tissue engineering. Besides presenting solubility in most of common solvents, PLGA can take different shapes and sizes, and encapsulates molecules of all sizes. Usually, higher rates of PGA (which contains methyl site groups) lead to a higher hydrophobicity and degradation of the polymer [75]. On the other hand, lactide-rich PLGA are less hydrophilic and degrade more slowly, since it absorbs less water [74]. Moreover, physical properties of PLGA are known to vary depending on different factors such as the molecular weight, which plays an important role in the drug-loading capacity on the polymer surface [76].

#### 3.1.4. Chitosan (CS)

IONPs have also been coated with CS. This material is a natural, long-chain polymer, generated by the combination of 2-amino-2-deoxy-β-d-glucan with glycosidic linkages, which can be obtained by chitin deacetylation [77]. Its positive charge drives the CS carriers to the cell membrane (negatively charged) and its mucoadhesive properties extend the CS retention in the target sites, making it interesting for application in drug delivery systems [77,78]. Furthermore, CS is biocompatible, biodegradable and presents low toxicity [79,80,81].

Many CS-nanosystems have been developed over the last few years [82,83,84,85,86,87], relying on the aforementioned advantages and water solubility [88]. Coating of IONPs with this polymer does not change the thermal and magnetic properties of the nanoparticles, serving as support for drug binding [89]. Also, a one-pot synthesis in the presence of CS of low molecular weight showed that it was capable of protecting IONPs from aggregation due to the electrostatic repulsion between the positively charged nanoparticles [22].

This polymer, however, presents certain limitations as a coating material, mainly associated with the partial protonation of its amino groups in water at physiological pH, what reduces CS solubility [90,91]. To overcome such issues, chemical changes can be performed in order to make CS derivatives more water-soluble [91]. A practical example is the *O*-carboxymethyl CS, which uses hydrogen bonding between water and the polymer in combination with carboxyl group to obtain water solubilization [92]. Also, a polyelectrolyte complex of carboxymethyl starch-CS can be used as a coating for IONPs, producing stable, biocompatible and mucoadhesive nanosystems [93].

### 3.2. Organic Surfactants

Surfactants (e.g., oleic acid, lauric acid) are extensively used to functionalize IONPs [54], mainly when synthesized in organic solutions. IONPs coated with dimercaptosuccinic acid (DMSA) have an anionic surface, which avoids opsonization and clearance by the reticuloendothelial system, with consequent reduction of cell toxicity [25,70]. Oleic acid and trisodium citrate are also capable of stabilizing nanoparticles by creating repulsive forces (mainly steric repulsion) to balance the magnetic and van der Waals attractive forces [94].

However, the long hydrocarbon chains of the surfactants make the nanoparticles hydrophobic, hindering their application in vivo, and require measures to prevent or reverse this aspect [94,95]. Investigations to overcome these limitations led to experiments with surfactant-functionalized IONPs through hydrophobic interactions, demonstrating that lower values of surfactants’ critical micelle concentrations were associated with more efficient coating of the IONPs, with greater dispersion in solutions and lower nanoparticle clustering [24].

### 3.3. Inorganic Compounds

Some applications are favored in inorganic compound-containing nanosystems, such as catalysis, bioseparation, optical bioimaging and biological labeling [29,70]. Moreover, inorganic compounds have the potential to increase the antioxidant properties of bulk IONPs [33]. Silica, carbon, metals, oxides (metal and non-metal) and sulfides are the most widely tested.

SiO_2_ is a classical coating material for IONPs, since it has the capacity of enhancing the IONPs dispersion in solutions, and to turn them more stable and protected in acidic medium [11,90]. The silanol groups on their surface also offer perfect anchorage for ligands, providing various functional groups to the nanosystem [16,29,33,70]. Most of silica coatings also contribute to reduce the IONPs toxicity [26,96,97,98].

Carbon-based coatings have interesting features, such as chemical and thermal stability, good electrical conductivity, solubility, and serve as a barrier against IONPs oxidation, being useful for diverse applications [28,29]. As an example, novel nanosystem composed by magnetic carbon/Fe_3_O_4_ with nanoscale zero-valent iron was able to remove 99.7% of Pb(II) from aqueous solutions [99].

Metal coatings can also prevent IONPs oxidation owing to their low reactivity [70]. They can bind to IONPs via electrostatic linking, forming core-shell structures that can be modified according to their functional groups and surface charges [29,100]. In this regard, the electron transfer between silver and IONPs in a nanosystem creates a silver coating positively charged, allowing the conjugation of different antibiotics to the silver-decorated IONPs [100]. In addition, silver coating does not alter the magnetic properties of the original iron oxide powder. Finally, metal coatings can also suffer modifications with compounds such as thiol to enable their linkage with diverse biomolecules [101]. 

Oxide and sulfide are common in IONPs functionalization, in order to stabilize the nanosystem without interfering with its magnetic features [11]. For metal oxides, it is possible that the combination of two different magnetic phases creates a new composite with pronounced magnetic properties [11]. The nanosystem can be formed by oxidation of the outer shell of the nanoparticles or it can just be additionally deposited [29]. The choice of a coating for the IONPs must take into account their intrinsic properties and the purpose of the nanosystem. For instance, ZnO was selected as the most appropriate compound for an anticancer nanosystem due to both its intrinsic anticancer properties and biocompatibility [102].

### 3.4. Bioactive Molecules

In this category are included bioactive structures such as lipids, peptides, and proteins [29,70,103]. In IONPs-based nanosystems functionalized with peptides, the biomolecules are able to maintain the stability of the nanostructures, as well as the magnetic properties of the IONPs [27,104]. 

Human and bovine serum albumin (HSA and BSA) are also used in biomedical and pharmaceutical applications, and can be attached to IONPs by desolvation [105]. BSA-coated IONPs has a negatively charged surface that avoids electrostatic interactions with negative biological elements such as plasma and blood cells, therefore maintaining IONPs stability [106].

While for matrix-dispersed structures IONPs are distributed in a matrix, thus preventing aggregation, in shell-core-shell they are confined between two functional materials [28,29]. In turn, Janus particles possess two compartments, one being the IONPs with magnetic properties and the other, composed by different functional molecules [107].

## 4. Drugs Bound to IONPs

**Figure 2:**
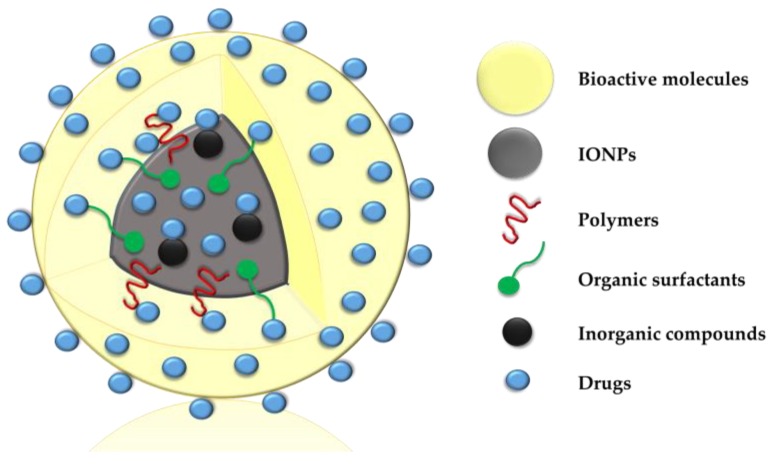
Schematic illustration of drugs directly bound to iron oxide nanoparticles (IONPs) or to core-shell nanosystems.

Magnetic nanoparticles have greater reactive area and ability to cross biological barriers than their micrometric counterparts, which favors their use in drug delivery systems. In this context, different classes of drugs can be directly bound to IONPs or to core-shell nanosystems, as shown in Figure 2. Such binding can occur by adsorption, dispersion in the polymer matrix, encapsulation in the nucleus, electrostatic interactions and covalent attachment to the surface [5,108], aiming to improve their pharmacological properties. IONPs have been used as carriers of anticancer, alternative, immunosuppressive, anticonvulsant, anti-inflammatory, antibiotic and antifungal agents.

### 4.1. Anticancer Drugs

One of the major challenges in cancer treatment is the tolerance developed throughout the therapy, which reduces response to the medicament. When a drug is bound to IONPs, this tolerance can be overcome due to a reduction of the “efflux pumps” that transport the drug to the outside of the cell [109], what ultimately promotes an increase in drug concentration in cancerous tissues [109]. IONPs-based nanocarriers are also able to reduce undesired interactions with other molecules [110] and toxic effects on normal tissues [111]. Furthermore, these nanosystems are less specific than molecules such as antibodies and peptides, thus acting on various types of cancer [111].

Over the last two years, the main anticancer drug coupled to IONPs was doxorubicin (DOX) [112,113,114,115,116,117,118,119,120,121,122,123,124,125,126,127,128,129,130,131,132,133,134]. This drug can be covalently bound to functionalized IONPs [123] or establish electrostatic interactions with negatively charged groups present in the magnetic nanocarriers [133]. A nanosystem with mesoporous hydroxyapatite (HA)-coated IONPs for DOX release was able to incorporate 93% of DOX from a concentration of 5 ppm [112]. In 24 h, only 10% of the DOX was released at pH 7.4, while for pH 5.5 it reached a release of 70%, making IONPs-DOX-HA a favorable nanosystem for the treatment of acidic solid tumors [112].

Current investigations have also synthesized IONPs employing different shells with anticancer agents conventionally administered, such as β-cyclodextrin [135], carmustine [136], cetuximab [137,138,139], cytarabine [140], daunomycin [141], docetaxel [142,143], epirubicin [144], 5-fluorouracil [145,146,147,148,149,150,151,152,153], gemcitabine [22,154,155,156,157,158], methotrexate [159,160,161], mitoxantrone [162,163,164,165] and paclitaxel [27,104,166,167,168,169,170,171,172]. It is noteworthy, however, that these nanosystems demonstrate magnetic properties only in the presence of external magnetic fields to prevent agglomerations of nanoparticles [111] and to allow a satisfactory performance in the target sites.

### 4.2. Alternative Drugs

Alternative drugs may also be linked to nanosystems. Curcumin, a natural polyphenolic hydrophobic compound from *Curcuma longa* rhizomes, has been incorporated into IONPs for preventing and fighting cancer [10,106,133,173,174,175]. It is believed that this herbal product remains captured at the hydrophobic interface between components of surfactant-stabilized nanocarriers [133]. In this sense, Unterweger et al. [176] proposed the use of photodynamic therapy associated with the hypericin photosensitizer (a component of the plant *Hypericum perforatum* or St. John’s wort, with antitumor properties), IONPs and dextran, concluding that this combination is a promising alternative for cancer treatment. Hypericin was covalently coupled to dextran-coated IONPs by glutaraldehyde, and the particles of this nanosystem were able to induce the death of cancer cells, besides not presenting agglomeration even after 12 weeks of storage in water [176].

Another active component of herbal origin used to fight cancer cells is berberine, which exhibits poor bioavailability in tumor sites due to the lack of hydrophilicity. To address this limitation, this compound was linked to IONPs and Sanazole, and the obtained nanosystem displayed a reducing effect on hypoxic tumor volume in mice [177]. Finally, essential oils have been bound to IONPs to improve their stabilization and reduce their volatility. Such nanostructures can be incorporated on the surface of catheters [178] or into wound dressing materials [103] in order to decrease microbial adherence and biofilm-associated infections.

### 4.3. Immunosuppressives

High concentrations of immunosuppressants may generate serious secondary complications in patients with transplants or autoimmune diseases [179]. To minimize this problem, a nanosystem composed by silica (SI)-coated IONPs was formulated to act as a carrier of mycophenolic acid (MPA), the main component of the immunosuppressive mycophenolate mofetil [180]. MPA was bound to the SI-coated IONPs by means of hydrophobic interactions, and the resulting nanosystem was biocompatible at the concentration of 0.56 mg/L, with capacity of transporting up to 30% of MPA’s weight. At this concentration, the IONPs-SI-MPA nanosystem was able to reduce the secretion of human interleukin 2 and tumor necrosis factor-α, indicating the activation of immune cells [181]. Despite the 10-fold lower MPA supply when compared to other studies [181,182], this nanosystem promoted a similar efficacy in cytokine upregulation.

### 4.4. Anticonvulsants

Nanotechnology has also generated a novel and non-invasive approach for the treatment of temporal lobe epilepsy associated with pharmacological resistance. A nanosystem composed by anti-interleukin-(IL)-1β monoclonal antibody (1-β mAb) covalently attached to IONPs functionalized with PEG was developed and injected into the caudal vein of rats with acute temporal lobe-induced epilepsy [183]. The MRI revealed a greater number of IONPs-anti (IL)-1β mAb-PEG in the epileptogenic tissues with respect to IONPs alone and the control group (saline solution), demonstrating a higher neuroprotective effect of the nanosystem. The effect of SI-coated IONPs loaded with the antiepileptic phenytoin (PHT) was also analyzed in an in vivo model assessing drug resistant convulsions [184]. IONPs were previously coated with SI to avoid oxidation and improve stability. Thereafter, PTH was bound to SI-coated IONPs via adsorption, forming a nanosystem capable of carrying around 250 μg of PHT per 100 mg of nanoparticles [184]. After administration of 3-mercaptopropionic acid (3MPA) in rats to induce convulsions associated with P-gliprotein cerebral overexpression [185], the IONPs-SI-PHT nanosystem was able to significantly increase the afterdischarge threshold values compared to the group of rats receiving pure saline solution, indicating its potential to reduce the neural excitability and the incidence of convulsion [184].

### 4.5. Anti-Inflammatories

The hydrophobic anti-inflammatory Ketoprofen (KTF) was encapsulated with IONPs, polypyrrole (PPL) and PEG to determine its release profile [186]. For nanoparticles without PEG, the encapsulation efficiency of 20% KTF’s weight was 98%, ratifying the high binding capacity of the drug, which had its complete release after three hours in phosphate-buffered saline solution. Electrostatic interactions between the positively charged PPL and the negatively charged KTF probably facilitated encapsulation, while PEG stabilized the IONPs by simple adsorption [186]. Jia et al. [187] coupled IONPs and hyaluronic acid (AH) to a furan-functionalized dexamethasone peptide (GQPGK), aiming to target the system to adipose tissue defect sites. This dexamethasone peptide was attached to the nanostrutures via covalent bonds, and the IONPs-AH-GQPGK nanosystem presented the desired adipogenic effect on co-culture of human adipose derived stem cells, evidencing great potential for application in adipose regeneration therapies. In turn, IONPs also showed potential for acting as carrier of prednisolone to the cochlea. Intratympanic administration of these nanoparticles in conjunction with prednisolone did not promote severe injury in rats, which highlights the possibility of developing alternative nanocomposites for the treatment of auditory disorders [188].

### 4.6. Antibiotics

The emergence of highly resistant bacterial strains and the reduced alternatives to conventional antibiotics has aroused interest in the design of antibiotic-carrier nanosystems. IONPs functionalized with CS have been used as carriers of streptomycin [189,190]. As a physical mixture, this antibiotic showed a rapid release (20 min) in phosphate-buffered saline, while in the form of a nanosystem its full release was completed only after 350 min, indicating the ability of IONPs to act in controlled-release systems [189].

Different groups of antibiotics (rifamycin, anthracycline, fluoroquinolone, tetracycline and cephalosporin) were bound by physical adsorption to silver nanoparticles (Ag)-loaded IONPs [100]. It is believed that Ag (positively charged) interacts electrostatically with the IONPs, allowing linking of these antibiotics [100]. Similarly, rifampicin, doxycycline, cefotaxime, and ceftriaxone were also attached to IONPs decorated with Ag, and the mechanisms of association varied for each type of antibiotic, including electrostatic binding with negative sites and hydrogen bonding [191]. Synthesis using the green sonication-assisted procedure was tested for the conception of the nanosystem IONPs-Ag-rifampicin [192]. The adsorption of rifampicin was evaluated at different Ag concentrations (5.3, 7.7, 10.1, 15.1 mass%) in the IONPs-Ag nanocomposite, and the lowest and highest adsorption occurred for Ag at 5.3 and 7.7 mass%, respectively [192]. 

Other models of therapeutic resources have also been studied, including IONPs-ciprofloxacin nanosystem [193], which may be functionalized with lactose particles [194]. Furthermore, nanosystems composed by amikacin, amoxicillin, bacitracin, cefotaxime, erythromycin, gentamicin, kanamycin, neomycin, penicillin, polymyxin, streptomycin and vancomycin directly coupled to IONPs (i.e., without involving a shell coating) have been investigated [195].

### 4.7. Antifungals

Fungal diseases are opportunistic infections that often affect immunocompromised patients and, if not properly treated, can be fatal. Since Nystatin (NYS) is one of the most commonly used fungicides, Hussein-Al-Ali et al. [196] prepared a nanosystem composed by IONPs, CS and NYS. The authors demonstrated that the release profile of NYS in a physical mixture of these isolated compounds lasted about 20 min, compared to 1800 min of the IONPs-CS-NYS nanosystem. This difference can be explained by the electrostatic interaction between NYS (negatively charged) and CS (positively charged), by which it can be inferred that the IONPs-CS-NYS nanosystem was able to generate a controlled release of NYS. Ketoconazole and amphotericin B are other antifungal drugs that have been tested in the development of magnetic nanosystems in order to decrease their side effects and improve antifungal action. Ketoconazole was coupled to epoxy-functionalized IONPs immobilized with HSA, and its binding mechanism occurred via hydrophobic interaction [197], while amphotericin B was directly bound to IONPs by a reaction between amine and aldehyde groups, respectively from the antifungal drug and IONPs [198].

## 5. Antimicrobial Activity of IONPs-Based Nanosystems

**Figure 3:**
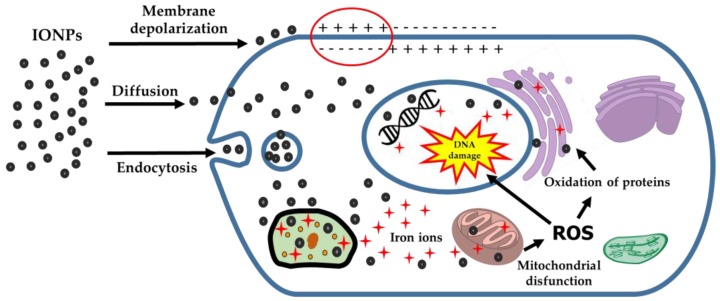
Main mechanisms of action by which systems based on iron oxide nanoparticles (IONPs) generate cell toxicity. ROS: reactive oxygen species.

Studies suggest that the potential of magnetic nanoparticles to generate microbial toxicity is due to a series of interactions, including membrane depolarization with consequent impairment of cell integrity [199], production of reactive oxygen species (ROS) with lipid peroxidation and DNA damage [200], and release of metal ions that affect cellular homeostasis and protein coordination [201] (Figure 3).

The antimicrobial potential of nanosystems has been evaluated against microorganisms in the planktonic state or forming biofilms, whose stage of development can affect the nanoparticles’ activity. The minimum inhibitory concentration (MIC) of the IONPs-amoxicillin nanosystem on *Staphylococcus aureus* and *Escherichia coli* planktonic cells was shown to be 3 to 4 times lower than the antibiotic alone [202]. This nanosystem was also able to decrease the initial adhesion of those bacteria to polystyrene during the first 24 h of biofilm formation. In turn, the IONPs-CS-streptomycin nanosystem exhibited a more significant effect on Gram-negative microorganisms than on Gram-positive [189,190].

Aiming to potentiate antibiotics such as rifampicin, doxycyclin, ceftriaxone and cefotaxime, studies proposed their linking to 1.6% Ag-decorated IONPs [100,191]. While IONPs alone did not prevent the growth of *Bacillus pumilus*, small inhibition halos (≤2 mm) were observed for IONPs-Ag, IONPs-Ag-ceftriaxone and IONPs-Ag-cefotaxime nanosystems [100]. Furthermore, halos around 20 mm were found for IONPs-Ag-rifampicin and IONPs-Ag-doxycyclin [100]. For *S. aureus*, only the IONPs-Ag-rifampicin, IONPs-Ag-doxycycline and IONPs-Ag-ceftriaxone nanosystems were effective, producing inhibition halos ranging from 10 to 24 mm [191]. Furthermore, the IONPs-SI-Ag-vancomycin nanostructure was more effective than free vancomycin in inactivating strains of *E. coli* and methicillin-resistant *S. aureus* [203]. Differences in the employed antibiotic concentrations may help to explain the results obtained. Finally, higher silver concentrations (5–10%) attached to IONPs and rifampicin were able to impair the growth of *Streptococcus salivarius* and *S. aureus*, but the same trend was not found for *Pseudomonas fluorescens* and *B. pumilus*, indicating that the effect of the nanocarrier is species-dependent. The increase in Ag concentration also improved the antibacterial properties against *S. salivarius* and *S. aureus*, amplifying the action spectrum of the antibiotic.

The incorporation of IONPs-based nanosystems into medical devices has shown promising results regarding the inhibition of microbial colonization. Catheter surfaces coated with essential oils-loaded IONPs decreased initial cell adhesion of *S. aureus* and *Klebsiella pneumoniae*, with a lesser effect on more mature stages of biofilm formation [178]. In turn, coating of textile fibers of wound dressings with patchouli essential oil-attached IONPs was effective in reducing the number of *S. aureus* biofilm cells [103]. Still concerning natural compounds, gallic acid-coated IONPs was shown to exhibit similar antimicrobial effects compared to ampicillin, streptomycin and nystatin against *E. coli*, *Bacillus substilis* and *Aspergillus niger*, respectively [204].

Despite the positive outcomes described above, conflicting results were reported for other nanosystems. While a nanosystem composed by IONPs, spray-dried lactose (SDL) and ciprofloxacin was shown to decrease the total biomass and metabolic activity of *Pseudomonas aeruginosa* biofilms (with an enhanced effect promoted by a magnetic field) [194], the IONPs-ciprofloxacin nanosystem did not exhibit antimicrobial effects against *E. coli*, *P. aeruginosa*, methicillin-sensitive *S. aureus*, methicillin-resistant *S. aureus*, *Streptococcus pneumoniae*, vancomycin-sensitive *Enterococcus faecalis*, vancomycin-resistant *E. faecalis*, *Acinetobacter baumannii*, *Proteus mirabilis*, *K. pneumoniae*, *Streptococcus pyogenes*, *Haemophilus influenzae*, *Staphylococcus epidermidis*, *Enterobacter aerogenes*, *Citrobacter freundii*, and *Enterobacter cloacae* tested in the planktonic state or forming biofilms [193]. Unfavorable results were also reported for *P. aeruginosa*, in which stimulation of biofilm formation was promoted by IONPs [205,206].

Drug carriers have also been tested as alternatives to combat the resistance of oral biofilms to commercially available drugs. Chlorhexidine (CHX), an antimicrobial agent used to control oral biofilms, was shown to be more effective when bound to IONPs in reducing biofilm biomass of *S. aureus*, *E. faecalis* and *Candida albicans*, in comparison with the drug applied alone [207]. Moreover, CHX particles functionalized with IONPs inhibited the growth of *Porphyromonas gingivalis* and, when incorporated into HEMA-UDMA resin discs, revealed a CHX release kinetics influenced by magnetic field [208].

It is believed that nanoparticles have the ability to adsorb and penetrate into biofilms due to their physicochemical characteristics, such as surface charge, hydrophobicity and high surface area ratio by volume [209,210]. Positively charged and neutral IONPs promoted higher reduction of cells of *Streptococus mutans* biofilms with respect to negatively charged counterparts [211], highlighting the influence of the surface properties of magnetic nanoparticles on their antibiofilm activity. Furthermore, IONPs have a good performance as catalysts, being nominated as catalytic IONPs (IONPs-CAT) or nanozymes. IONPs-CAT in association with H_2_O_2_ showed an enhanced antimicrobial effect on *S. mutans* biofilms compared to its counterpart without H_2_O_2_ in vitro [212]. Under in vivo conditions, IONPs-CAT attenuated caries initiation and the severity of lesions, while H_2_O_2_ alone did not exhibit considerable effects [212], showing the potential of the IONPs in combating dental caries.

**Figure 4:**
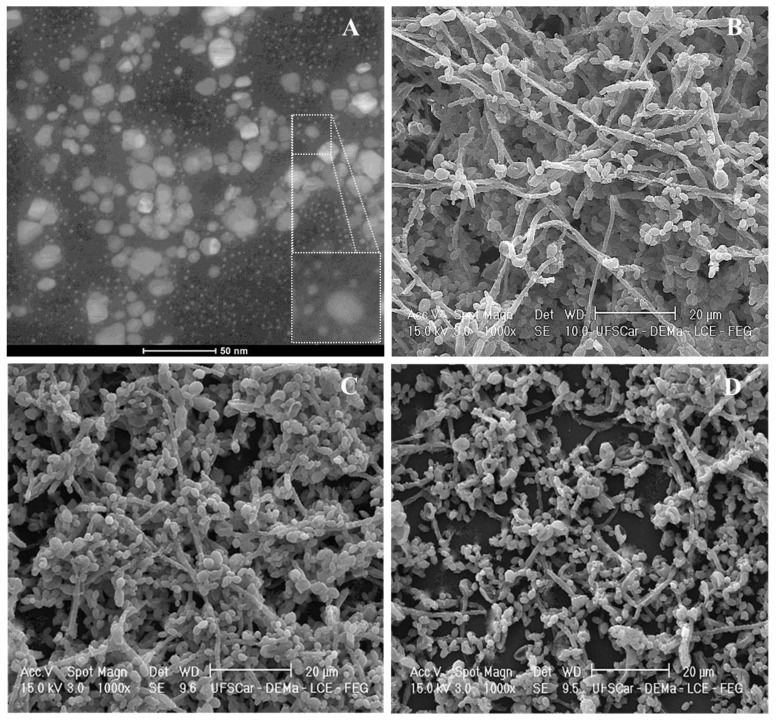
(**A**) Transmission electron microscopy image obtained from a miconazole (MCZ)-carrier nanosystem based on iron oxide nanoparticles (IONPs) and chitosan (CS); increased image at the bottom right corner shows the core of a CS-coated IONP, with MCZ particles adhered to CS; (**B**) Scanning electron microscopy (SEM) image of untreated dual-species biofilm of *C. albicans* and *C. glabrata* (48 h). SEM images of dual-species biofilms treated for 24 h with 78 µg/mL MCZ (**C**) or MCZ-containing nanosystem at 78 µg/mL (**D**). Source: the authors (unpublished data).

Besides drugs with antibacterial action, the association of IONPs with antifungal agents has shown to promote a significant effect on different microbial species. When amphotericin B and NYS were coupled to IONPs, the antifungal effect of the resulting nanosystems was potentiated in comparison with the free drugs on *Candida spp.* [198]*.* In addition, CS-coated IONPs loaded with NYS promoted reductions of 1.3, 35.0, 99.0 and 99.9% in the number of cultivable cells of *S. aureus*, *E. coli*, *P. aeruginosa* and *C. albicans*, respectively, despite free NYS promoted a greater inhibition halo for *C. albicans* compared to the IONPs-CS-NYS nanosystem and the IONPs alone [196]. Miconazole (MCZ), another conventional antifungal agent used for the treatment of oral candidiasis, has also been incorporated in CS-coated IONPs (Figure 4A). This new nanosystem showed a higher ability in disrupting dual-species biofilms of *C. albicans* and *C. glabrata* (Figure 4D) with respect to MCZ alone in vitro (Figure 4C), as evident by a less compact structure composed by a lower number of cell layers partially covering the surface (Figure 4D).

## 6. IONPs Toxicity

Conflicting evidence regarding the toxicity of IONPs has been reported in in vitro and in vivo studies [213,214,215,216]. Factors inherent to nanosystems involving IONPs tend to directly interfere with their toxicity. For instance, changes in nanoparticle size and shape were shown to play an important role on cell toxicity, with rod-shaped or nano-sized IONPs being more toxic than sphere-shaped and micrometric particles, respectively [217]. The configuration of the nanosystem can also influence IONPs toxicity. A Janus microsphere encapsulating mesenchymal stem cells (MSC) and IONPs in two different compartments was proven to facilitate the magnetization and movement of the microspheres (due to the higher load capacity of IONPs), and to reduce cell toxicity, since the IONPs’ derived toxic chemicals were isolated from the MSC compartment [107]. Furthermore, the surface charge of IONPs may affect cell cytotoxicity and genotoxicity. Positively charged IONPs were shown to be more toxic, since they undergo nonspecific interactions and adsorptive endocytosis with the negatively charged cell membrane, thus increasing their intracellular accumulation and affecting cell membrane integrity [218]. Other factors such as concentration, type of coating, form of administration, as well as the cell line may explain the different results for IONPs toxicity [21,22,23,25,26,66,71,72,96,97,98,163,214,215,219,220,221,222,223,224,225,226,227,228,229,230,231,232,233,234,235,236,237,238,239,240,241,242,243,244,245,246,247,248], as shown in Table 1.

### 6.1. Mechanisms of IONPs Toxicity

The toxicity of IONPs for different cell lines may be partially explained by the production of ROS, which causes cellular oxidative stress [249]. When uptaken by cells via endocytosis, IONPs tend to accumulate in the lysosomes and are degraded in iron ions (Figure 3). In theory, the ions could cross the membranes and reach regions such as the cell nucleus and mitochondria, reacting with hydrogen peroxide and oxygen, thus generating ROS [250,251] (Figure 3).

Despite oxidative stress is the most well-studied hypothesis of toxicity and cell damage, iron overload caused by exposure to IONPs can also generate serious deleterious effects and lead to cell death [246,250,251]. On the other hand, magnetite was shown to be responsible for increasing the level of lipid peroxidation and decreasing antioxidant enzymes of human lung alveolar epithelial cells (A-549), displaying a concentration-dependent toxicity in vitro [252]. In addition, a high dose of IONPs (with consequent iron excess) promoted elevated lipid metabolism, breakage of iron homeostasis and exacerbated loss of liver functions, being considered a risk factor for cirrhosis in a mice-model study [253].

### 6.2. Influence of Coatings on IONPs Toxicity

The surface coating of IONPs is a widely used procedure in order to make these nanoparticles biocompatible and non-toxic, supposedly due to lower number of oxidative sites, with consequent less DNA damage [254]. In this sense, a well referenced in vitro study showed that uncoated IONPs produced greater toxicity for rat fibroblast cells when compared to nanoparticles coated with polyvinyl alcohol, due to changes in the protein functions and cellular ion balance caused by gas vesicles after exposure to uncoated particles [255]. Additionally, uncoated IONPs were shown to increase intracellular density (greater ROS production), which might lead to relevant morphological cellular alterations, whereas protein-coated IONPs reached densities capable of causing tolerable changes [256]. Also, the use of lauric acid, protein corona of BSA, or dextran as shells for IONPs did not promote genotoxic effects on human granulosa cells [257].

Coating with polymers and essential oils can also reduce the toxic effects of IONPs. PLGA-functionalized IONPs were able to reduce the destructive effects on lysosomes, mitochondria, golgi body and endoplasmic reticulum compared with uncoated nanoparticles, which induced the cells to autophagy [258]. In turn, IONPs functionalized with patchouli essential oil promoted low cytotoxicity on mammalian cells and good biodistribution after intraperitoneal injection in mice [103].

Despite the remarkable advantages of IONPs coating, some divergences have been reported in the literature. Previous data showed that the coating of IONPs with D-mannose or poly-l-lysine was not able to prevent their toxicity in murine neural stem cells, as these nanoparticles demonstrated negative effects on the mithocondrial homeostasis [259]. This emphasizes the importance of conducting additional tests other than cell viability for a more complete assessment of the toxic effects of these nanosystems.

## 7. Conclusions and Perspectives

Microorganisms resistant to conventional treatments evolve faster than the creation of new drugs and antibiotics. Within this context, IONPs bear great potential for use in nanosystems capable of overcoming the physical barriers of the microbial biofilm matrix in delivering the drugs directly to the target. The next steps consist in further exploring the magnetic properties of IONPs to improve the drug effect using lower concentrations, thus reducing side effects and toxicity.

The various methods of synthesis have allowed the creation of nanoparticles with different sizes, structures, dispersions and surface modifications. However, as wide variations have been reported among different research protocols, a direct comparison of the results obtained cannot be done. This aspect points out to the need for a refinement/standardization of protocols of synthesis and functionalization prior to in vivo testing, aiming to produce nanoparticles with adequate stability, size-control, biocompatibility and bioavailability.

Despite the growing body of scientific evidence on the use of IONPs in drug delivery systems, not all relevant drugs of medical/dental interest have been investigated, either alone or in combination with IONPs. In this sense, the conception of novel IONPs-based nanosystems able to carry multiple drugs simultaneously, and with adequate release control on the target tissues could be beneficial in several clinical situations. These include the prevention/control of diseases associated with multiple microorganisms (e.g., bacteria and fungi), as well as conditions that require different categories of drugs (e.g., anti-inflammatories, antibiotics and antifungals).

Finally, regarding the applicability of IONPs-based nanosystems, most of clinical trials conducted so far have focused on MRI, so that clinical assessment of applications other than MRI is expected in the near future. For such purpose, large and industrial-scale production of IONPs-based nanosystems is an important challenge to be overcome.

## Figures and Tables

**Table 1:** Summary of studies assessing the toxicity of iron oxide magnetic nanoparticles (IONPs) carried out between 2014 and 2018.

Reference	Year	Coating	Concentration	Cell Line or in Vivo Model	Study Model	Toxicity
[221]	2014	Manganese (Mn)	5, 10, 20, 50 and 100 mg/L (in vitro); 150 µmol/kg Fe/kg body weight (in vivo)	Murine Balb/3T3 fibroblasts (in vitro) and CD1 female mice (8 weeks old) (in vivo)	In vitro/In vivo	Dose-dependent toxicity
[222]	2014	Poly(lactic-*co*-glycolic acid) (PLGA) + 5-Fluorouracil	50, 100 and 200 µΜ	Human prostate cancer cell line DU145	In vitro	Non toxic
[26]	2014	Silica shell (Fe_3_O_4_/SiO_2_ NPs)	0.5, 1, 2.5 and 5 nM	A549 and HeLa cells	In vitro	Silica coating diminished Fe_3_O_4_ cytotoxic and genotoxic effects
[21]	2014	Poly-(ethylene glycol) (PEG) (PEGylated IONPs)	100 and 500 ppm Fe	Bovine vascular smooth muscle cells (VSMCs)	In vitro	PEGylated IONPs showed less cytotoxicity than uncoated IONPs
[223]	2014	-	1, 3 and 5 mg/mL	Epithelial cell and cancer cell lines; ECR 116 (NCBI code: C570)	In vitro	Non toxic
[224]	2014	Aminodextran (AD), 3-aminopropyltriethoxysilane (APS) and dimercaptosuccinic acid (DMSA)	0.05, 0.1, and 0.5 mg/mL	HeLa (human cervical adenocarcinoma)	In vitro	Non toxic
[225]	2015	-	214 mg/L	Neuronal cell line (Rat pheochromocytoma-PC12 cells)	In vitro	Neurocytotoxic
[226]	2015	-	25, 50, 75 and 100 mg/L	Human hepatoma cells (Hep G2)	In vitro	Toxic (reduced cell viability with oxidative damage)
[227]	2015	Polyhydroxybutyrate (PHB)	29–500 μM	MCF-7, SKBR-3 and HeLa human breast and ovarian cancer cell lines	In vitro	Non toxic
[228]	2015	Curcumin (Cur)	IONPs: 120 mg/L Cur: 40 mg/L	Wild type MDKC and human neuroblastoma cells	In vitro	Non toxic
[229]	2015	L-DOPA (L-3,4-dihydroxyphenylalanine)	0–0.05 mg/mL (in vitro) and 2.5 mg/mouse (approximately 125 g/kg body weight) (in vivo)	Normal mouse L929 fibroblasts/C57BL/6 mice	In vitro/in vivo	Non toxic
[230]	2015	Polyacrylic acid (PAA) and non-coated	4, 20 and 100 mg/L	Human T lymphocytes	In vitro	Non genotoxic
[231]	2015	-	200 and 400 mg/L (in vitro), and 200 mg/L (in vivo)	Mouse fibroblast cell (in vitro) and wistar rat’s liver and kidney (in vivo)	In vitro/In vivo	Non toxic
[232]	2015	Alginate (Alg)/Alg + D-galactosamine (GA)	0–1000 mg/L	Liver cancer/hepatocellular carcinoma (HepG2) cell line	In vitro	Non toxic
[233]	2015	Uncoated (U-Fe_3_O_4_) and oleate-coated Fe_3_O_4_ (OC-Fe_3_O_4_)	10.8, 21.6 and 108 mg/L	Human lymphoblastoid TK6 cells and primary human blood cells	In vitro	U-Fe_3_O_4_ was not toxic; OC-Fe_3_O_4_ was cytotoxic in a dose-dependent manner and genotoxic
[71]	2015	Bare (uncoated) SPION (BS) and PEG (PEG-SPION (PS))	50.8 mg/kg b w for PS and 16.3 mg/kg b w for BS	BALB/c Swiss Albino mice	In vivo	PEGylation reduced the toxicity of BS (Low toxicity)
[234]	2015	Cobalt	75, 150, 250, 500, 750 and 1,000 mg/L	MCF-7 cell lines	In vitro	Moderate toxicity to cancer cells
[235]	2016	Rhamnose	0,1,2, 5, 10, 25, 50 and 100 µg Fe mL^−1^ for cancer cell lines, and 15.63 to 1000 µgFemL^−1^ for fibroblasts cell lines	Human glioblastoma cell lines (T98G and U251MG) and the human urinary bladder carcinoma cell line (ECV304), mouse fibroblast (BALB/3T3) cell line and its clone (A31-1-1).	In vitro	Moderate toxicity to tumoral cell lines and non toxic to fibroblast cells
[96]	2016	Silica and oleic acid	5–300 mg/L	Human neuroblastoma SHSY5Y and glioblastoma A172	In vitro	Low citotoxicity/oleic acid-coated IONPs with less citotoxicity than silica-coated IONPs
[163]	2016	Mitoxantrone (MTO)	0.0001–0.1 mg/L	Human primary tubular epithelial cells (hTEC)	In vitro	Moderate toxicity (depends on the drug loaded to the SPION)
[25]	2016	2,3-dimercaptosuccinic acid (DMSA)	15, 30, 60 e 80 mg/L (IONPs)	human mesenchymal stem cells from dental pulp tissues	In vitro	Non toxic
[236]	2016	No coating and curcumin-coating	1–1000 mg/L	Human umbilical vein endothelial cells (HUVECs)	In vitro	Curcumin-coated IONPS were less toxic than uncoated IONPs
[237]	2016	Polyacrylic acid-*co*-maleic acid (PAM) + tissue plasminogen activator (tPA)	30 μg Fe/mL	Human umbilical vein endothelial cells (HUVECs)	In vitro	Low toxicity
[238]	2016	Polymer (converted from Poly(lactic-*co*-glycolic acid) nanoparticles)	0.005–0.32 mg/mL	SKOV3 human ovarian cancer cells and NIH/3T3 murine fibroblasts	In vitro	Low toxicity
[22]	2016	Chitosan + Gemcitabine	IC_50_ for SKBR-3 (4.8 µM) and MCF-7 (1.5 µM)	SKBR-3 and MCF-7 breast cancer cells	In vitro	More cytotoxic to the tested breast cancer cell lines than free gemcitabine
[239]	2016	-	10, 25, 50, 75, and 100 mg/L	Human peripheral lymphocytes	In vitro	Moderate toxicity
[215]	2016	-	0–1000 mg/L	Human whole blood cultures	In vitro	Dose-dependent toxicity
[240]	2016	-	65 ng/mL (in vitro), and 520 µg Fe_3_O_4_/kg and 20.8 µg Fe_3_O_4_/kg (in vivo)	Mouse embryonic fibroblasts NIH3T3 (in vitro) and Wistar rats (in vivo)	In vitro/In vivo	Non toxic at a desirable concentration
[23]	2016	PEG350 and PEG2000	50–200 mg/L (in vitro)/12.5, 25 and 50 mg/kg/day (in vivo)	Monkey kidney ephitelium (Vero), dog kidney fibroblasts (MDKC) and mouse embryonic fibroblast (NIH-3 T3) (in vitro) and Swiss albino male mice (in vivo)	In vitro/In vivo	SPION-PEG2000 showed no toxicity in vitro, but lead to liver and kidney injury in vivo. In vitro, SPION-PEG350 showed no toxicity up to 100 µg/mL
[241]	2016	c(RGDyK) + dopamine	1.50, 2.07, 2.87, 3.97, 5.49, 7.59 and 8.50 g/kg	Kunming mice of SPF grade	In vivo	Non toxic
[242]	2017	Poly-(ethylene glycol) (PEG) and polyethylenimine (PEI) polymers + folic acid (FA-IONPs) Doxorubicin + FA IONPs (DOX@FA-IONPs)	0.2–10 mg/L	MCF7 cells	In vitro	FA-IONPs show low cytotoxicity and DOX@FA-IONPs is more cytotoxic than free DOX
[243]	2017	Tri-block copolymer: poly(ε-caprolactone)-poly(ethylene glycol)-poly(ε-caprolactone) (PCL-PEG-PCL, PCEC)	0, 0.5, 1, 2, 5 and 10%	NIH 3T3 cells	In vitro	The PCEC coating reduced Fe_3_O_4_ NPs toxicity
[97]	2017	SiO_2_ (Fe_m_O_n_-SiO_2_ composite and SiO_2_-Fe_m_O_n_ core-shell IONPs)	0.7, 7.0 and 70.0 µg	Human umbilical vein endothelial cell culture (cultured HUVECs)	In vitro	Dose-dependent toxicity in the presence of silica. Bare IONPs were less toxic
[244]	2017	Chitosan (CS) + calf-thymus DNA (DNA)	-	Human foreskin fibroblast cell line (HFFF2)	In vitro	Non toxic
[245]	2017	Zinc/Cobalt	10, 100, 250 and 500 μM	Primary human bone marrow-derived mesenchymal stem cells (hMSCs) and human osteosarcoma-derived cells (MG-63)	In vitro	The levels of toxicity do not compromise the biocompatibility
[246]	2017	-	10, 25, 50, 100, and 200 mg/mL (0.3 mL/egg in airspace)	Fertilized eggs of White leghorn (Gallus gallus domesticus)	In vivo	Neurotoxic in lower doses and 100% mortality at 200 mg/mL dose
[72]	2017	Polyvinylpirrolidone	1, 10, 25, 50 and 100 μg/mL	Human neuroblastoma (SH-SY5Y cell line)	In vitro	Dose-dependent toxicity
[98]	2018	Luminescent ruthenium (II) complex encapsulated with silica shell + amine group (APTMS)-Fe_3_O_4_@SiO_2_@[Ru(Phen)_3_] 2+@SiO_2_@NH_2_	10, 50 and 100 μg/mL	Cancer cell (B16F10) and normal cell (CHO)	In vitro	Low cytotoxicity
[66]	2018	-	0.1, 0.5, 1, 2.5, 5 and 7.5 mg/mL	MCF7 and 3T3 cell lines	In vitro	Dose-dependent toxicity
[219]	2018	Polyethylenimine (PEI) and polyethylene glycol (PEG)	3.125–100 µg/mL (in vitro)/Up to 5mg/kg (in vivo)	RAW264.7 macrophages and non-phagocytic SKOV-3 ovarian cancer cells (in vitro)/SKOV-3 tumor bearing nude mice and BALB/c mice (in vivo)	In vitro/In vivo	PEI-coated-IONPs were toxic in vitro with dose-dependent toxicity in vivo/PEG-coated-IONPs presented low toxicity
[247]	2018	Polyamidoamine (PAMAM) dendrimer (Fourth generation—G_4_)	*Acute toxicity:* 25, 50 and 100 mg/kg *Chronic toxicity:* 0.5, 1, 5 and 10 mg/Kg	BALB/c mice	In vivo	Acceptable toxicity
[248]	2018	Chitosan (CS)-dextran (DX)	1, 5, 10, 50, and 150 µg/mL	Rat C6 glioma, human U87 glioma, and human cervix carcinoma HeLa cells	In vitro	Dose and time-dependent toxicity
